# N-terminal-pro brain natriuretic peptides in dogs and cats: A technical and clinical review

**DOI:** 10.14202/vetworld.2017.1072-1082

**Published:** 2017-09-18

**Authors:** Gabriela Vieira de Lima, Felipp da Silveira Ferreira

**Affiliations:** 1 Cardiology Service from the Pet du Bosque Veterinary Clinic, Cuiabá, Mato Grosso, Brazil; 2 Department of Veterinary Cardiology of the Qualittas Postgraduate Institute, Campinas, São Paulo, Brazil

**Keywords:** cardiac biomarkers, cats, congestive heart failure, dogs, NT-proBPN

## Abstract

Biomarkers are quantitative indicators of biological processes performed by an organ or system. In recent years, natriuretic peptides (NPs) have emerged as important tools in the diagnosis and therapeutic monitoring of heart diseases. Research has shown that serum and plasma levels of N-terminal pro brain NP (NT-proBNP) in dogs and cats are the only biomarkers that afford to diagnose and monitor congestive processes and, indirectly, the myocardial function of small animals. The present review discusses the peer-reviewed specialized literature about NT-proBNP and presents and compares the potential clinical applications of this NP in veterinary medicine of small animals, considering diagnosis, follow-up, and prognosis of myocardial or systemic diseases. The relevance of NT-proBNP is associated with sample stability, easy determination in laboratory, sensitivity, accuracy, and the possibility to analyze myocardial function. These advantages are specially important when NT-proBNP is compared with other cardiac biomarkers, mostly those that indicate the integrity of the myocardial cell. Fast NT-proBNP assays are marketed today and may be used in association with complementary tests. Together, these methods are an important source of information in differential diagnosis of heart and lung diseases as well in the early diagnosis of cardiopathy in dogs and cats, proving valuable tools in treatment and prognosis.

## Introduction

Biomarkers are quantitative indicators of biological processes performed by an organ or system. They are important tools in the evaluation of normal, pathological, and pharmaceutical intervention processes, providing information about diagnosis, extension of lesions, and prognosis. Ideally, a biomarker has to be highly sensitive so as to afford early detection of low titers, as in the onset of a disease. High specificity is also very important since a biomarker has to be detectable in a specific organ or tissue. In addition, low cost, easy quantification, and an appropriate interval between infection and the detection of antibodies are other characteristics of a suitable biomarker [1-4].

In recent years, natriuretic peptides (NP) have emerged as important tools in the diagnosis and therapeutic monitoring of heart diseases. The main NPs today are the atrial NP (ANP), the brain NP (BNP), and the c-type NP, which take part in cardiovascular and cardiorenal homeostasis. More specifically in veterinary medicine, the N-terminal fragment of BNP (NT-proBNP) is the most used NP currently [5,6].

Research has shown that serum and plasma levels of NT-proBNP in dogs and cats are the only biomarkers that afford to diagnose and monitor congestive processes and, indirectly, assess the myocardial function of small animals [2]. For this reason, the interest in NT-proBNP in veterinary medicine has grown in the past 10 years, even though it is used mainly in some research contexts because proper methodologies have not been validated and standardized for clinical practice [7-9].

In this scenario, several studies have been published about biomarkers, their uses, and results they indicate. Therefore, the present study discusses the peer-reviewed specialized literature about NT-proBNP and presents and compares the potential clinical applications of this NP in veterinary medicine of small animals, considering diagnosis, follow-up, and prognosis of myocardial or systemic diseases.

## What are Biomarkers?

A biomarker is a biochemical element that can be measured to evaluate the progression of a disease or the outcomes of treatment. Routinely used in several specialties, biomarkers are convenient tools in oncology, neurology, cardiology, pneumology, systemic inflammatory diseases, and metabolic conditions [3,10].

Cardiac biomarkers belong to four groups, which are used in various settings: Myocardial lesion or necrosis (cardiac troponins), myocardial function (NPs), homeostasis of serum lipoproteins (high-density lipoproteins and low-density lipoproteins), and inflammation of the cardiovascular system (C-reactive protein). However, in veterinary medicine, the last two types are not specific to myocardial diseases [4,7,11]. Moreover, troponins are more effective to evaluate ischemic lesions, which do not mirror the reality of veterinary cardiology. In other words, NPs are the only biomarkers that can be used to assess myocardial function, which is affected more commonly by primary structural cardiopathies in animals, such as dilated and hypertrophic cardiomyopathy (HCM), as well as myxomatous degeneration of valves [12,13]. However, high levels of these peptides in conjunction with the increase in cardiac troponin concentrations may indicate ischemic disease, like acute myocardial infarction, which may predispose to heart failure [14].

One of the essential characteristics of every biomarker includes the specificity for the organ studied, that is, the biomarker should not be released by other tissues. Furthermore, the biomarker should be released in proportion to the extension or severity of the disease or lesion, not affecting sample stability and providing the clinician with the necessary elements to choose and monitor the correct therapeutic protocol. The ideal biomarker is inexpensive and should be easily available for purchase. Some biomarkers are routinely employed in veterinary medicine, such as urea and creatinine in the evaluation of renal function, while cardiovascular biomarkers are still little used [13,15,16].

## The NP

NPs are a family of hormones with a 17-amino acid sequence clustered as a ring and stabilized by a cysteine bridge with two terminal portions, one with the carboxyl radical and one with an N-terminal amine group that play comparable roles in the cardiovascular system. Structurally, NPS are similar, though they differ in the biological effects they have. The main roles of NPs are associated with the regulation of homeostasis of the intravascular volume and the control of systemic pressure [17,18].

As a rule, NPs are produced and secreted in specific cardiac sites in response to myocardial overload and cardiomyocyte strain; yet, they are also released by other tissues (Table-1) [2,16,19].

**Table-1:** Types of NP and release sites based on Baisan *et al*. [16].

NP types	Release
ANP	Atrial
BNP	Mainly ventricles
CNP	Brain, endothelium, kidney, chondrocytes, and pituitary gland
DNP	Venom gland of *Dendroaspis angusticeps* (eastern green mamba)
VNP	Primitive heart, normally in fish

Together, NPs balance the effects of the renin–angiotensin–aldosterone system (RAAS), which is hyperactive during heart disease, promoting cardiac remodeling, and inducing congestive heart failure (CHF). Furthermore, NPs antagonize this effect by promoting natriuresis, blood flow in kidneys, diuresis, and vasodilation, increasing the heart’s diastolic function, although they also influence vascular permeability and inhibit the growth of smooth muscle cells [15,20-22].

BNP is secreted as a precursor molecule and is cleaved by serum proteases to form identical amounts of active C-terminal fragments (C-BNP) and one inactive NT-proBNP. However, C-BNP has a very short half-life (approximately 90 s), which may be an obstacle in the determination of circulating concentrations. On the other hand, NT-proBNP levels are determined more easily, since they have a comparatively long half-life (about 120 min) and are more stable during sample collection and handling [15,23,24].

The clearance of NPs is an important regulator of plasma concentrations of these hormones. The process takes place through two pathways. The first is an enzymatic degradation by neutral endopeptidase and the second is a receptor-mediated endocytosis followed by lysosomal degradation through the NP receptor C. In the first, the degradation occurs through a neutral endopeptidase, which cleaves NPs in several inactive fragments. The main clearance organs are the liver, lungs, and, more importantly, kidneys [5].

## The BNP

This hormone was originally isolated from pig brain, although the BNP form circulating in healthy animals may be produced by ventricles. In a pathological scenario, BNP production is substantially high in ventricles, and it is cleaved inside ventricular cardiomyocytes. After cleavage BNP generates C-BNP, which promotes vasodilation and diuresis by binding to specific receptors in vascular and renal tissues in the effort to balance the effects of RAAS. It is involved in the regulation of the homeostasis of body fluids and blood pressure in response to stress or strain of the myocardium due to increased volume. The release of BNP seems to be volume dependent, and has natriuretic, diuretic, and hypotensive action, though the concentration of circulating BNP is lower than that of ANP since BNP levels are detectable only in the presence of a change in heart anatomy [19,22,25-32].

The gene responsible for the codification of BNP is located in the short arm of chromosome 1, which generates a precursor hormone, prohormone BNP (pre-pro-BNP). The pre-pro-BNP is formed by 134 amino acids, 26 of which are signaling amino acids that are swiftly removed. The resulting product is the pro-BNP, formed by 108 amino acids. Two proteolytic enzymes, furin and corin, cleave pro-BNP into NT-proBNP, a terminal fragment formed by 76 amino acids with no biological activity, and a biologically active fragment, BNP, which is formed by 32 amino acids. As a rule, the depuration of BNP is attributed to an internalization carried out by NP and excretion through urine, and its half-life is also influenced by proteolytic cleavage [5,16,22,29,33].

## Laboratory Evaluation

The laboratory evaluation of NPs requires special methods to collect blood. Most importantly, it is necessary to delay degradation (like hemolysis, for instance) during collection procedures and transportation to a laboratory. Furthermore, samples have to be centrifuged and kept under refrigeration immediately after collection [5,9,31,34-37]. Sample storage methods also greatly influence the values of NT-proBNP in serum. For example, the degradation of the peptide by proteases during storage reduces NT-proBNP levels in canine serum. In this sense, one manufacturer of the NT-proBNP assay for dogs warns that plasma samples collected in EDTA should be centrifuged and mixed with a protease inhibitor immediately after collection. In cats, a protease inhibitor was shown to improve the stability of NT-proBNP in samples of plasma stored at room temperature [31].

The dose of ANP may be evaluated using immunoassays developed for humans due to the fact that its chemical structure is highly conserved in mammals. In turn, BNP is considered species-specific, which means that it is necessary to validate enzyme-linked immunosorbent assay (ELISA) assays to determine NT-proANP and NT-proBNP in dogs and cats [4,38,39].

## The Applications of BNP in Clinical Practice

The evaluation of heart disease in animals poses a challenge to veterinarians, since signs may be unspecific. Moreover, the diagnosis of heart murmur sometimes does not indicate the severity of a lesion, and any concomitant lung injury may influence the interpretation of confirmatory tests. Other diagnosis methods such as echocardiography and electrocardiograms are more specific, but they may not be readily available at the moment of examination in some settings [13,35].

Several studies were conducted using various approaches to determine whether high NT-proBNP values afford accurate diagnosis of a given disease. Nevertheless, NT-proBNP levels above the upper cutoff should never be used as a sole parameter; rather, NT-proBNP quantification is indicated concomitantly with other diagnosis methods such as echocardiography, radiography, electrocardiography, medical record, and evaluation of clinical signs [40].

NPs are widely used as biomarkers of cardiac function in humans. In recent years, they have also been employed in the diagnosis and prognosis of diseases in dogs and cats. It is believed that since NPs are secreted after strain and tension in myocardium, high NP levels in plasma may indicate the severity of heart disease in several species [13].

However, obstacles to the clinical use of NPs include physiological and pathological factors associated with cardiac function, which may directly affect NP concentrations and not indicate clearly the occurrence of a pathological process. For this reason, various authors have looked into ways to adjust individual reference and cutoff values concerning misdiagnosis of processes in diseases [13,41,42].

Based on the hypothesis that NP levels vary across dog breeds (in addition to the presence of a cardiac condition), Sjöstrand *et al*. [42] investigated the difference in NT-proBNP levels in plasma of 535 healthy animals of nine breeds. The authors observed significant differences in NP concentrations, and the interquartile range, especially of NT-proBNP, was wide for several breeds. Labrador retriever and Terra Nova were the breeds with the highest NT-proBNP concentration medians, which were 3 times as high as those observed for Dachshunds. Furthermore, plasma NP levels (especially NT-proBNP) varied considerably across breeds. Yet, further studies are necessary to establish specific reference values for each breed.

In the effort to shed light on the relationship between NT-proBNP and specific diseases affecting a given breed, Couto *et al*. [43] compared plasma NT-proBNP levels in 24 Greyhounds and 29 dogs of other breeds. It was shown that Greyhounds present functional heart murmur, relative cardiomegaly, and high levels of cardiac troponin I compared with other breeds. It was also reported that Greyhounds had higher NT-proBNP serum levels than other breeds, when means were 945 and 632 mg/L, respectively. In view of the high values characteristic to Greyhounds, the authors observed that the level of NT-proBNP should be taken with caution in this breed, compared with values observed for others, since it is an important factor in the diagnosis of a cardiomyopathy.

In the effort to differentiate respiratory from cardiac and non-cardiac signs, guidelines have been published concerning the use of NT-proBNP assays in dogs and cats. These guidelines include: (i) Normal or reduced levels of NT-proBNP are more common in non-cardiac conditions, while high levels are typical of cardiopathies, (ii) in animals with asymptomatic heart disease, high NT-proBNP levels may be misdiagnosed as having non-cardiac causes, such as a respiratory condition, and (iii) NT-proBNP levels have to be analyzed in the context of clinical record, physical examination, and conventional diagnosis methods like imaging [6,44].

## Clinical Applications of NT-proBNP in Dogs

NT-proBNP may be used in the early evaluation of the risk of a dog presenting heart disease or heart failure, even when the only clinical sign is dyspnea. NT-proBNP levels above 900 pmol/L in dogs are not compatible with myocardial overload, but concentrations over 735 pmol/L in Dobermans indicate dilated cardiomyopathy (DCM). The low concentration of NT-proBNP suggests non-cardiac disease (Table-2) [6], while higher levels indicate CHF [15,16,28].

**Table-2:** NT-proBNP for heart disease in dogs and cats.

Dogs	Values	Cats	Values
Normal (low probability of heart disease)	<800 pmol/L	Normal	<50 pmol/L
High (high probability of heart disease)	800-1800 pmol/L	Asymptomatic heart disease	50-100 pmol/L
Diagnosed heart disease	>1800 pmol/L	Probable heart disease	101-270 pmol/L
CHF	>2700 pmol/L	CHF	>270 pmol/L

Adapted from Anjos *et al.* [6]. CHF=Congestive heart failure

Several authors have underlined the importance of NT-proBNP as a biomarker of cardiac and non-cardiac causes of dyspnea in veterinary medicine. NT-proBNP is useful in dyspnea when this is the main health condition in dogs requiring emergency care. Two changes are used to diagnose heart condition early or within a few months (Table-3) [27,29,36,38,43-62]. The same authors also claim that the biomarker is useful to evaluate the severity of injury also before the emergence of necrosis or ventricular dysfunction as well as during follow-up to therapeutic procedures [4,20,24,41,48,55].

**Table-3:** Different applications of BNP in the diagnosis of cardiac and non-cardiac conditions in dogs and cats.

Disease	Species	n	Method	Values observed	Reference
Cardiac and non-cardiac dyspnea	Feline	10	ELISA	Control: 265 pmol/L Observed: >270 pmol/L	Singletary *et al*. [45]
	Canine	291	ELISA	Control: 1.287 pmol/L Observed: 5.110 pmol/L	Fox *et al*. [46]
MMVD	Canine	352	ELISA	Control: 455.3 pmol/L Observed: 4.920 pmol/L	Wolf *et al.* [47]
	Canine	21	ELISA	Control: 687.5 pmol/L Observed: >2.298 pmol/L	Ruaux *et al*. [48]
Babesiosis	Canine	60	ELISA	Control: 246 pmol/L Observed: 4.106 pmol/l	Lobetti *et al*. [49]
Pulmonary hypertension secondary to MMVD	Canine	30	Serum biochemistry	Control: 373 pmol/L Observed: 1799 pmol/L	Atkinson *et al*. [27]
Cardiac and non-cardiac pleural effusion	Feline	40	ELISA	Control: 214 pmol/L (plasma) 322.3 pmol/L (pleural fluid) Observed: 360 pmol/mL (plasma) 1.332 pmol/L (Pleural fluid)	Humm *et al*. [50]
Cardiac and non-cardiac pleural effusion	Feline	21	ELISA	Control: 69 pmol/L Observed: 982 pmol/L	Hassdenteufel *et al*. [51]
Cardiac and non-cardiac pleural effusion	Feline	55	ELISA	Control: 213.3 pmol/L (plasma) 322.3 pmol/L (Pleural fluid) Observed: 678 pmol/mL (plasma) 719 pmol/L (pleural fluid)	Hezzell *et al*. [52]
DCM	Canine	155	ELISA	Control: 457 pmol/L Observed: 1.143 pmol/L	Singletary *et al*. [44]
Persistent truncus arteriosus	Canine	40	ELISA	Control: 663 pmol/L Observed: 895 pmol/L	Hariu *et al*. [53]
Hypertrophic cardiomyopathy	Feline	78	ELISA	Control: 7.0 fmol/mL Observed: 854.1 fmol/mL	Connolly *et al*. [54]
Hypertrophic cardiomyopathy	Feline	40	ELISA	Control: 21 pmol/L Observed: 134 pmol/L	Hsu *et al*. [38]
Hypertrophic cardiomyopathy	Feline	35	ELISA	Control: 40 pmol/L Observed: 45 pmol/L	Singh *et al*. [55]
Hidden heart disease	Feline	113	ELISA	Control: 24 pmol/L Observed: 186 pmol/L	Fox *et al.* [56]
Hidden heart disease	Feline	146	ELISA	Control: 48 pmol/L Observed: 522 pmol/L	Machen *et al*. [57]
Visceral leishmaniasis	Canine	18	ELISA	Control: 800-1800 pmol/L Observed: 1346.0 pmol/L	Silva *et al*. [58]
PS	Canine	41	ELISA	Control: 485 pmol/L Observed: 3.000 pmol/L	Kobayashi *et al*. [29]
Right-sided congestive heart failure	Canine	67	ELISA	Control: 510 pmol/L Observed: 6.554 pmol/L	Kanno *et al*. [36]
Parvovirus infection	Canine	13	ELISA	Control: 0.05-1.4 pg/ml Observed: 2.68-30.1 pg/ml	Cenk and Mahmut [59]
Conditions affecting healthy retired Greyhounds	Canine	53	ELISA	Control: 1000 pmol/L Observed: Greyhounds: 946 pmol/L Non-Greyhounds: 632 pmol/L	Couto *et al*. [43]
Cardiotoxicity	Canine	80	ELISA	Control: 68.5-512.08 ng/ml Observed: 211.34 ng/ml	Crivellente *et al.* [60]
Pre-capillary pulmonary hypertension	Canine	20	ELISA	Control: 744 pmol/L Observed: 2.011 pmol/L	Kellihan *et al.* [61]
Chronic pulmonary hypertension	Canine	6	ELISA	Control: 445 pmol/L Observed: 1.049 pmol/L	Hori *et al.* [62]

ELISA=Enzyme-linked immunosorbent assay, DCM=Dilated cardiomyopathy, MMVD=Myxomatous mitral valve degeneration

Klüser *et al*. [39] evaluated the sensitivity of biomarkers and other diagnosis methods to predict sudden death in Dobermans with terminal DCM. The authors included NT-proBNP concentrations in an additional statistical analysis only when urea and creatinine levels were normal, as a way to avoid using misleadingly high levels of the fragment. The longitudinal prospective study included 95 Doberman Pinschers with DCM. 40 dogs died within 3 months of the last heart examination, and NT-proBNP values were compared with those measured in 54 dogs of the same breed that survived 1 year into the study. The authors concluded that NT-proBNP levels have high predictive value, but the strong correlation of the biomarker with diastolic volume in the left ventricle indicates that it may actually be a suitable alternative parameter when echocardiography is not available. NT-proBNP levels may be used to predict the increase in heart volume and, indirectly, assess survival based on the damage caused.

Boswood *et al*. [63] used the cutoff value of 201 pmol/L NT-proBNP to evaluate any statistically significant difference between dogs with cardiopathy and those with primary respiratory disease. In total, 77 dogs were included, 32 of which had cardiopathy with CHF, 28 had cardiopathy without CHF, and 17 had pneumonia only. All 32 dogs with cardiopathy and CHF were submitted to echocardiography, 30 of which were also submitted to thoracic radiography. 27 of the 28 dogs with heart disease and CHF were examined using echocardiography and 24 of which also underwent thoracic radiography. The 17 dogs with lung disease were submitted to thoracic radiography, 5 of which also underwent echocardiography. The study showed that NT-proBNP levels above the cutoff value may be used to discriminate patients with heart disease from those with respiratory injury, with good accuracy.

More recently, Fox *et al*. [46] analyzed the sensitivity and specificity of second-generation commercial NT-proBNP ELISA kits to differentiate cardiac from non-cardiac dyspnea. The authors examined 291 animals distributed in four groups (39 healthy animals, 74 animals with asymptomatic heart disease, 104 with the respiratory disease with or without associated heart disease, and 74 symptomatic animals with CHF). The results showed that the kits used afford to accurately differentiate the causes of dyspnea, with 87% specificity and 77% sensitivity. Furthermore, the authors observed that NT-proBNP levels are higher than 2,247 pmol/L in dogs with a heart condition.

In a recent study, Winter *et al*. [64] evaluated the variation in NT-proBNP values in dogs with different stages of myxomatous mitral valve degeneration (MMVD). The authors included 38 dogs (10 with MMVD Stage B1, 10 with MMVD Stage B2, and 8 with MMVD Stage C-stable). 10 healthy dogs were included as a control. The second-generation canine marker Cardiopet proBNp, produced by IDEXX, was used. NT-proBNP levels were measured in varied time frames (every hour, once a day, and once a week). Mean NT-proBNP in the control group was 543 pmol/L, while in the MMVD group the value was 991 pmol/L, varying within the 24-4086 pmol/L intervals.

MMVD is the cause of approximately 75% of all heart diseases in dogs. However, results of various studies are inconsistent concerning the reliability of serum levels of NPs to detect subclinical MMVD and differentiate it from the asymptomatic stages of the disease. With that in mind, Wolf *et al*. [47] included 116 healthy dogs and 236 dogs with MMVD in a study that used cutoff values of 1207 pmol/L for NT-proBNP and 1578 pmol/L for NT-proANP. The animals with MMVD with and without CHF could be differentiated with specificity of 85% and 86%, respectively. The results showed that levels of the NPs evaluated were high; in other words, they can be used as biomarkers in the scenario considered.

Oyama *et al*. [65] examined 119 dogs with MVD, 8 with DCM, and 40 healthy control animals to assess the efficiency of NT-proBNP to identify heart disease and evaluate its severity. Serum NT-proBNP levels were substantially high in the dogs with heart disease, compared with the control group. The authors used the cutoff value of 445 pmol/L to group healthy and sick dogs. The sensitivity and the specificity of the assay for heart disease were 83.2% and 90%, respectively.

Ruaux *et al*. [48] also investigated NT-proBNP values in nine dogs with MMVD of various degrees of severity. 12 healthy dogs formed the control group. Samples were collected weekly during 7 weeks. NT-proBNP levels were considerably higher in the dogs with the disease (2298 pmol/L), compared with the healthy animals (697.5 pmol/L), and varied significantly across animals with MMVD. The authors concluded that serial NT-proBNP quantification every 6 months may provide useful information for prognosis, helping differentiate healthy and diseased dogs and showing once again that NT-proBNP is a reliable biomarker in diagnosis and follow-up.

Importantly, NT-proBNP has always been investigated for its potential to detect CHF in dogs with asymptomatic DCM. The first commercial kits used to detect NT-proBNP had cutoff value of 3000 pmol/L, which did not provide the required reliability of results, casting doubt on the actual usefulness of the assay. The second-generation kits have cutoff values between 3000 pmol/L and 10,000 pmol/L. Such high cutoff values help diagnose dyspnea and primary heart disease — and even its severity — since serum levels of NT-proBNP increase with heart diseases [15,26].

In addition to establishing new cutoff values for NT-proBNP assays, Fox *et al*. [46] assessed the results obtained in their study considering the guidelines issued by the American College of Veterinary Internal Medicine, which classified heart disease based on stages of severity. Stage A included cases of patients predisposed to heart disease with no detectable structural heart conditions. Stage B included asymptomatic heart disease and was divided in B1 when patients showed no evidence of cardiac remodeling in radiographs and echocardiographs, and B2, when these exams revealed increased the left heart volume. In turn, Stage C included patients with CHF signals and associated structural heart conditions. Stage D represented the terminal stage of heart disease. No animal in Stage D was included in the study, but NT-proBNP levels were higher in dogs in Stages C and B2 than in B1 and A, respectively.

Singletary *et al*. [44] evaluated changes in serum levels of NT-proBNP as an indicator of hidden DCM (HDCM) and estimate survival in Dobermans. The authors examined 155 asymptomatic dogs based on the diameter of the left ventricle during systole using echocardiographs and/or the results of the Holter monitor during 24 h. The cutoff value of NT-proBNP was 457 pmol/L. Of the 155 dogs included, 73 were diagnosed with HDCM. When increased NT-proBNP values occurred concomitantly with changes in Holter readings, sensitivity was 94.5%, and specificity was 87.8%, with 91.0% accuracy. The presence of over 50 premature ventricular complexes in the 24-h Holter monitoring, the detection of high NT-proBNP levels, or both parameters indicated that a patient had an 87.3% chance of having HDCM. The change in NT-proBNP levels observed in that study afforded to distinguish Dobermans in Stage II or III of DCM from healthy counterparts and the dogs with high levels of the fragment presented poor prognosis compared with those with lower concentrations. The results also showed that, when used together with other diagnosis procedures, NT-proBNP is reliable both in the early diagnosis of HDCM and in the estimation of survival.

Hariu *et al*. [53] investigated the relationship between NT-proBNP and persistent arterial duct (PAD) in 10 dogs with the disease and 30 healthy individuals. The animals with PAD were submitted to ductal occlusion surgery, and NT-proBNP levels were evaluated again after the procedure. Serum NT-proBNP levels measured before surgery were significantly higher in dogs with PAD (mean: 895 pmol/L, in the 490-7,118 pmol/L interval), compared with the control group (663 pmol/L; 50-1,318 pmol/L). On the 90^th^ day after occlusion, NT-proBNP levels dropped considerably (597 pmol/L; 154-1,858 pmol/L). Left atrium size decreased significantly after 24 h and after 90 h. The authors concluded that the use of NT-proBNP in surgical follow-up in dogs with PAD is an important tool to evaluate post-operative outcome.

Pulmonary stenosis (PS) is a common congenital cardiopathy in dogs and may be classified in three types, namely, subvalvular, valvular, and supravalvular. Kobayashi *et al*. [29] carried out a retrospective study to investigate the clinical applications of plasma NT-proBNP levels in dogs with PS, as in the assessment of the severity of the disease, for example. The authors examined 23 asymptomatic and 7 symptomatic beagles with PS and 11 healthy counterparts as a control. The cutoff value was 764 pmol/L. Compared with healthy dogs, NT-proBNP levels were significantly higher (485 and 3,000 pmol/L, respectively).

Kanno *et al*. [36] investigated the applicability of serum NT-proBNP levels to assess the signs of right-sided CHF using 16 healthy dogs and 51 symptomatic and asymptomatic dogs with the disease but given no treatment. Mean NT-proBNP levels were 510 pmol/L in healthy dogs, below the values found for symptomatic (6,554 pmol/L), and asymptomatic (1,654 pmol/L) animals. These results suggest that plasma levels of NT-proBNP increase sharply in dogs with right-sided CHF, making it the most useful biomarker for the disease.

In a study that compared serum levels of NT-proBNP, NT-proANP, and cTnI in eight control dogs with respiratory disease but without pulmonary hypertension (PH) and 12 dogs with precapillary PH, Kellihan *et al*. [61] found higher mean NT-proBNP levels in dogs with PH (2,011 pmol/L, in the 274-7713 pmol/L interval) than in the control group (744 pmol/L, 531-2710 pmol/L). The authors concluded that NT-proBNP levels are significantly higher in dogs with precapillary PH compared with dogs with respiratory disease without PH and that this parameter is useful to predict severity of PH. However, NT-proBNP levels and cTnI levels do not increase in dogs with precapillary PH.

The specialized literature also reports the possibility to use NT-proBNP in cases of infectious myocarditis secondary to babesiosis in dogs. In an interesting study, Lobetti *et al*. [66] maintain that myocarditis may pose a challenge in diagnosis since the general signs of acute myocardial dysfunction such as dyspnea and weakness are common also in canine babesiosis, which makes the identification of symptoms more difficult. The authors claim that cardiac biomarkers may be useful to determine whether a dog with babesiosis also presents myocarditis. They included 45 animals positive for babesiosis and compared the results to those of 15 healthy animals. The dogs were divided into groups according to clinical status: (i) Mild disease without complications, (ii) severe disease without complications, (iii) severe disease with complications, and (iv) control group. Mean NT-proBNP values for these groups were 246, 650, 638, and 106 pmol/L, respectively. Importantly, the three groups with babesiosis had statistically higher levels of the biomarker, compared with the control group. The study concluded that babesiosis induces an increase in NT-proBNP concentrations due to the effects of the disease on the myocardium and that these levels are proportional to severity.

In a study that evaluated the changes in cardiac biomarkers and coagulation profiles in dogs with parvovirus, Cenk and Mahamut [59] included 27 naturally infected and six healthy animals. The objective was to assess the importance of these biomarkers in the prognosis of parvovirus. The authors concluded that NT-proBNP levels were higher in dogs with the disease, showing that the biomarker is useful to determine prognosis based on the magnitude of the increase in serum levels.

Furthermore, Silva *et al*. [58] looked into biomarkers of myocyte lesions and stress in 18 dogs naturally infected with visceral leishmaniasis with and without symptoms. Mean NT-proANP, NT-proBNP, cTnI, and CK-MB levels were 1,138 pmol/L (875-1175 pmol/L), 1,160 pmol/L (803-2,304 pmol/L), 0.22 ng/mL (90.15-0.51 Ng/mL), and 116.7 U/L (113-222 U/L). All values of specific cardiac biomarkers were high, compared with the normal interval previously determined for dogs. The authors concluded that the quantification of specific cardiac biomarkers, including NT-proBNP, is useful to identify a myocardium lesion secondary to visceral leishmaniasis.

## BNP in the Diagnosis of Heard Disease in Cats

As in dogs, commercial NT-proBNP kits are also used in emergency veterinary medicine to diagnose the main cause of dyspnea in felines, especially in the task of prescribing the best therapeutic protocol [21].

Singletary *et al*. [45] evaluated the effectiveness of NT-proBNP to differentiate the causes of feline dyspnea in 10 cats. The authors evaluated clinical record, imaging exams, and NT-proBNP levels, and concluded that quantification of the fragment is a highly sensitive tool in the differential diagnosis of dyspnea, helping define the best treatment.

In cats, NT-proBNP levels below 100 pmol/L are not associated with cardiomyopathy (Table-2). Yet, concentrations in the 100-270 pmol/L range indicate cardiomyopathy, though with no clinical changes. When values exceed 270 pmol/L, clinical signs of cardiomyopathy may be observed. The assay detects hidden cardiomyopathy with sensitivity levels between 86% and 100% and specificity in the 89-100% range [16,55].

Feline HCM is a primary myocardium disease characterized by concentric thickening of the left ventricular myocardium. It is caused by mutations in cardiac myosin that binds to C-protein, but it may also be idiopathic. HCM affects mostly Maine Coon and Ragdoll breeds. The disease may be diagnosed using NT-proBNP levels, but only at an advanced stage, when serum levels of the fragment are above the 100 pmol/L cutoff [55,67,68].

Hsu *et al*. [38] evaluated the efficacy of plasma NT-proBNP levels to diagnose subclinical HCM in 40 adult Maine Coon cats and mixed breeds thereof. Echocardiography findings of 9 cats were normal, 12 were suspected HCM cases, 9 had the mild form of the disease, and 10 indicated severe HCM. For the authors, measurements of NT-proBNP are highly sensitive and specific to detect severe HCM in cats, but not the mild manifestation of the condition. Therefore, the test cannot be used in the preventive diagnosis of the disease, since altered values are observed only in severe cases.

In a study, based on an univariate analysis with 41 cats with HCM, Borgeat *et al*. [69] observed that NT-proBNP levels above 250 pmol/L are associated with heart failure, but this association was not significant when compared with the clinical signs or size and efficiency of the left atrium. Yet, when considered in light of high cardiac troponin I (cTnI) levels (0.7 pmol/L cutoff), high NT-proBNP levels strongly correlated with poor prognosis in the Univariate analysis.

Connolly *et al*. [54] investigated the potential of plasma levels of NT-proBNP and NT-proANP to differentiate cats with heart disease and with or without associated CHF. The area under the curve (AUC) of the efficacy receptor curve was larger when NT-proBNP levels in serum were used to distinguish control cats from those with CHF. The second largest AUC was observed when NT-proBNP levels were used to distinguish healthy cats from the animals that were positive for heart disease. NT-proBNP levels were significantly higher than those of NT-proANP, indicating that the biomarker is useful in the early screening of HCM in cats with heart disease.

The accuracy of the point of care (POC) NT-proBNP ELISA to assess the probability of hidden heart disease from moderate to severe in cats suspected of heart disease was investigated by Machen *et al*. [57], in a study with 146 asymptomatic cats presenting heart murmur, gallop rhythm, arrhythmia, or cardiomegaly. The study population included 43 healthy cats and 50, 31, and 6 animals with mild, moderate, and severe hidden heart disease, respectively. No heart disease was diagnosed in 16 cats, which were considered suspected cases. The cutoff value used was 24-1500 pmol/L NT-proBNP, and 100 pmol/L was considered abnormal POC NT-proBNP ELISA differentiated cats with moderate and severe hidden heart disease with 83.3% sensitivity, 82.6% specificity, and 82.9% accuracy. The authors concluded that patients were positive in the POC NT-proBNP ELISA are at higher risk of moderate hidden heart disease, compared with negative cases.

Solter *et al*. [23] included 31 cats distributed in two groups: 18 healthy and 13 with cardiomyopathy in a study conducted to quantify C-terminal pro-BNP levels in feline plasma using ELISA. Levels of C-terminal pro-BNP varied between 1.7 and 78.8 pmol/L in 13 cats with cardiomyopathy and between 1.4 pmol/L and 1.8 pmol/L in 18 healthy counterparts. The authors concluded that C-terminal pro-BNP levels were lower than those of NT-proBNP and similar to those observed in humans and dogs with heart failure, two species in which low C-terminal pro-BNP levels have been associated with progression of the disease.

In a study carried out to differentiate cardiac and non-cardiac pleural effusion in cats, Hassedenteufel *et al*. [51] used 21 animals with the moderate and severe forms of the condition that were divided in two groups, with or without CHF. The authors measured NT-proBNP levels using a specific kit for felines, and the cutoff value adopted was 258 pmol/L. The felines with pleural effusion associated with CHF had higher NT-proBNP levels (355-1286 pmol/L), compared with those with pleural effusion only (26-160 pmol/L). NT-proBNP afforded to differentiate cats with cardiac and non-cardiac causes of pleural effusion.

Similarly, Humm *et al*. [50] analyzed the applicability of NT-proBNP to differentiate the origin of feline pleural effusion in a comprehensive study with three objectives: (i) To determine whether NT-proBNP may be detectable also in pleural lavage and urine of cats with effusion, (ii) to establish whether NT-proBNP levels in pleural lavage and urine correlate with the values of NT-proBNP in plasma, and (iii) to determine whether NT-proBNP in pleural lavage and urine can be used to distinguish the cardiac and non-cardiac causes of pleural effusion. The authors used 40 cats that underwent complete clinical examination and appropriate examinations, like echocardiography. The samples of pleural lavage were obtained by thoracentesis, and NT-proBNP levels were measured using a specific kit for feline samples. Blood samples were collected by venipuncture, while urine samples were obtained by cystocentesis or spontaneous collection in 28 of the 40 animals. The results indicate that NT-proBNP detected in pleural lavage may tell apart the cardiac and non-cardiac causes of pleural effusion, with precision values similar to those observed for plasma NT-proBNP. In contrast, urine was shown to be an unreliable indicator if heart disease causes the respiratory condition.

Hezzel *et al*. [52] compared the potential of first- and second-generation ELISA kits and fast NT-proBNP tests to differentiate pleural effusion associated or not with heart conditions. The authors used plasma and pleural lavage from 57 patients that were sorted as groups according to cause of pleural effusion (cardiac or non-cardiac). The second-generation ELISA afforded good accuracy in the diagnosis of de cause of the condition (plasma: 95.2% sensitivity, 82.4% specificity; pleural lavage: 100% sensitivity, 76.5% specificity). Concentrations of NT-proBNP were higher in the pleural lavage (719 pmol/L) than in plasma (678 pmol/L), which translates as the need to adopt different cutoff values for each material. The fast test presented good sensitivity (92.5%) and specificity (87.5%) for plasma samples. When pleural lavage was used, sensitivity was good, but specificity was low, 100% and 64.7%, respectively, showing that the accuracy of first- and second-generation assays was similar.

## NT-proBNP in Prognosis and Follow-up

Physical examination is a limited tool in terms of reliability to analyze the hemodynamic profile of patients CHF. However, plasma levels of NT-proBNP are strongly correlated with changes in lung capillary pressure, not only the ventricular filling pressure. Patients that do not respond positively to optimized vasodilation therapy may be identified using serum levels of NT-proBNP [20].

For Maisel *et al*. [70], NT-proBNP predicts the risks of death or CHF in asymptomatic patients and may be used as a complementary test in the evaluation of such risks and the short-term follow-up to CHF treatments. Plasma levels BNP correlate positively with the severity of heart disease or failure [6], and are useful as a prognostic tool. In the study conducted by Singletary *et al*. [44] with Dobermans, NT-proBNP was also used to predict survival, but increased levels indicated longer, or shorter survival of the patients included.

NT-proBNP levels are highly correlated with heart volume and rate in dogs with heart disease. A study with dogs with heart failure showed that NT-proBNP levels are significantly higher in animals with DCM that died within 60 days of follow-up (median: 4,865 pmol/L), indicating that high levels of the parameter signal poor survival [71].

Cardiovascular toxicity may be observed in preclinical and clinical events. Preclinical cardiotoxicity may be detected using biochemical methods (troponin or BNP) or histopathological (endomyocardial biopsy). BNP increases even when no signs of heart failure are present, which shows its high sensitivity to predict cardiotoxicity. Only a few studies have looked into the use of NPs and troponin in follow-up, but these works have shown that high serum levels of biomarkers, even of BNP, help identify cardiotoxicity during treatment [72,73].

One study with male 80 beagles, Crivellente *et al*. [60] investigated the cardiotoxic capacity of casopitant, an agonist NK1 receptor that was being developed to treat depression and anxiety in dogs and that is cardiotoxic when administered for long periods. The signs associated with the increase in cardiac troponin I due to the oral prescription of casopitant 40 mg/kg for 39 weeks included increased heart weight, myocardium necrosis, degeneration, and inflammation. However, the results obtained (Table-3) underscore the importance of NT-proBNP as a reliable, sensitive, and non-invasive tool in the early detection of cardiac hypertrophic changes in male Beagles in preclinical toxicological studies. The authors also observed that NT-proBNP may be used as a predictive biomarker of the pharmacological response during treatment of heart disease, as demonstrated in other investigations, and that it is an excellent tool in preclinical studies during the design of new drugs.

Finally, it should be highlighted that, although NPs remain an important element in the diagnosis of congestive processes, they do not afford the differential diagnosis of these conditions, and should be used in association with complementary exams, such as thoracic radiographs and ecodopplercardiograma [74].

## Conclusion

Cardiac biomarkers should be considered as important as any other biomarker, such as renal and hepatic ones. Nevertheless, due to the little knowledge, some veterinarians have of the topic, biomarkers are used mostly in research, or more specialized clinics.

The relevance of NT-proBNP is associated with sample stability, easy determination in laboratory, sensitivity, accuracy, and the possibility to analyze myocardial function. These advantages are specially important when it is compared with other cardiac biomarkers, mostly those that indicate the integrity of the myocardial cell, such as troponins and CM-MB.

Fast NT-proBNP assays are marketed today and may be used in association with complementary tests. Together, these methods are an important source of information in differential diagnosis of heart and lung diseases as well in the early diagnosis of cardiopathy in dogs and cats, proving valuable tools in treatment and prognosis.

## Authors’ Contributions

FSF and GVL conceptualized, designed, and wrote the manuscript, and contributed equally with literature analysis and manuscript revision. All authors read and approved the final manuscript.
